# Association of metformin administration after septic shock with short-term and long-term survival in septic shock patients with diabetes

**DOI:** 10.1186/s13613-025-01490-8

**Published:** 2025-05-21

**Authors:** Bo-Yeong Jin, Sukyo Lee, Woosik Kim, Jong-Hak Park, Hanjin Cho, Sungwoo Moon, Sejoong Ahn

**Affiliations:** 1 Department of Biomedical Sciences, Seoul National University College of Medicine, Seoul, Republic of Korea; 2 Department of Emergency Medicine, Korea University Ansan Hospital, 123, Jeokgeum-ro, Danwon-gu, Ansan-si, Gyeonggi-do 15355 Republic of Korea

**Keywords:** Septic shock, Type 2 diabetes mellitus, Metformin, Diabetic medication, Biguanide, Survival

## Abstract

**Background:**

In addition to glycemic control, the anti-inflammatory effects and protective effect of metformin on sepsis have been reported in animal studies, which may be beneficial for patients with septic shock. Few observational studies have evaluated metformin administration after sepsis or bacteremia; however, these studies did not specifically analyze septic shock or long-term outcomes. Therefore, this study aimed to evaluate the associations between metformin administration after septic shock and the short- and long-term survival in septic shock patients with type 2 diabetes mellitus.

**Method:**

This retrospective observational study used data from a prospectively collected sepsis registry. From October 2016 to June 2022, adult septic shock patients with type 2 diabetes mellitus were included in this study. The variable of interest was metformin administration within 48 h after diagnosis of septic shock. The 90-day mortality and 365-day mortality were evaluated as outcomes. A multivariable Cox proportional hazards model was conducted.

**Results:**

A total of 320 patients were included in the study. Metformin administration within 48 h after diagnosis of septic shock was associated with lower 90-day mortality (13.0% vs. 39.8%, *P* < 0.001), 365-day mortality (23.3% vs. 48.3%, *P* = 0.001), and in-hospital mortality (9.3% vs. 28.6%, *P* = 0.002) than those who did not administer metformin within 48 h. Metformin administration within 48 h was independently associated with decreased 90-day mortality (adjusted hazard ratio [aHR]: 0.371, 95% confidence interval [CI]: 0.153–0.900, *P* = 0.028) and 365-day mortality (aHR 0.453, 95% CI 0.219–0.937, *P* = 0.033) after adjusting for potential confounders. Similar results were found for metformin administration within 72 h after septic shock (aHR 0.433, 95% CI 0.235–0.797, *P* = 0.007 for 90-day mortality and aHR 0.450, 95% CI 0.264–0.767, *P* = 0.003 for 365-day mortality).

**Conclusions:**

In septic shock patients with type 2 diabetes mellitus, metformin administration within 48 h was associated with lower 90-day and 365-day mortality. While these findings suggest potential benefits of metformin administration after septic shock, further large, multicenter studies are warranted.

**Supplementary Information:**

The online version contains supplementary material available at 10.1186/s13613-025-01490-8.

## Background

Sepsis is a life-threatening organ dysfunction caused by a dysregulated host response to infection and is a major global burden with high mortality [1, 2]. Septic shock is the most severe form of sepsis, with a mortality rate of approximately 38% [2]. Comprehensive management of patients is required to reduce the high mortality due to septic shock [3].

The prevalence of diabetes mellitus in 2019 was estimated to be 463 million and is expected to increase globally [4]. Diabetes mellitus is a well-known risk factor for cardiovascular diseases and is associated with a high rate of mortality in various diseases [5]. Diabetes mellitus is associated with an increased risk of infection, higher sepsis-related mortality, and higher rate of colonization by resistant pathogens [6].

Metformin is a well-known first-line medication for type 2 diabetes mellitus [7]. Previous studies have reported that metformin is associated with reduced mortality in patients with diabetes mellitus in various conditions or diseases [8–10]. In addition to glycemic control, metformin has an anti-inflammatory effect and a protective effect in sepsis in animal studies [11–15]. The protective effects of metformin in sepsis have been observed not only in prophylactic but also in post-insult administration in various animal models. These effects have been reported in the heart, nervous system, liver, and other organs [16–21].

Metformin can be administered before sepsis or after sepsis during hospitalization. Most previous observational studies have evaluated pre-admission or pre-morbid metformin administration and mortality in sepsis [22–25]. However, observational studies to evaluate metformin administration after sepsis during hospitalization [26] or during bacteremia [27] are limited. Further those studies did not specifically analyze the effects of metformin administration on septic shock and its long-term outcomes. Therefore, this study aimed to evaluate the association between metformin administration after septic shock and the short- and long-term survival in septic shock patients with type 2 diabetes mellitus. We hypothesized that metformin administration after septic shock is associated with reduced short- and long-term mortality in patients with septic shock and type 2 diabetes mellitus.

## Methods

### Study design and setting

This retrospective observational study used data from a prospectively collected sepsis registry. This study was conducted at Korea University Ansan Hospital, the only tertiary academic teaching hospital in Ansan-si with 700,000 residents [10]. The Korea University Ansan Hospital has 880 beds, including 44 intensive care unit beds. The study was conducted in accordance with the principles of the Declaration of Helsinki. This study was approved by the Institutional Review Board of Korea University Ansan Hospital (2022AS0266). The requirement for informed consent was waived by the Institutional Review Board owing to the observational design of this study.

### Study population

Adult patients (age > 18 years) with type 2 diabetes mellitus who were diagnosed with septic shock on the day of hospital admission between October 2016 and June 2022 were included in this study. Patients with a do-not-resuscitate (DNR) order, patients with chronic kidney disease, and those whose 90-day mortality data were unavailable due to loss to follow-up were excluded. Patients who survived for < 48 h were excluded because metformin administration within 48 h would not be possible and including data of those patients might result in survival bias.

### Definitions and data collection

Sepsis was defined as an acute increase from baseline in total sequential organ failure assessment (SOFA) score ≥ 2 due to infection [1]. Septic shock was defined as a serum lactate level > 2 mmol/L and the requirement of vasopressors despite adequate fluid resuscitation to maintain a mean arterial pressure ≥ 65 mmHg. All patients were managed according to the Surviving Sepsis Campaign guidelines [3].

The variable of interest was metformin administration within 48 h after diagnosis of septic shock. The other variables evaluated were other diabetic medications within 48 h, and metformin administration within 72 h (3 days) after diagnosis of septic shock. Since alpha-glucosidase (*n* = 1), incretin (*n* = 0), meglitinides (*n* = 1), sodium glucose cotransporter 2 (SGLT2) inhibitors (*n* = 0), and thiazolidinedione (*n* = 3) were administered to only a few patients, hence we evaluated only sulfonylureas, dipeptidyl peptidase 4 (DPP4) inhibitors, and insulin as other diabetic medications. Diabetic medications administration within 48 h after septic shock was confirmed using electronic medical records recorded by the physicians and nurses.

Acute kidney injury (AKI) was defined according to the Kidney Disease Improving Global Outcomes guidelines as stages 1–3 [28]. High-dose vasopressor was defined as the requirement of norepinephrine-equivalent dose ≥ 0.25 µg/kg/min. Low-dose vasopressor was defined as the requirement of norepinephrine-equivalent dose < 0.1 µg/kg/min. Cardiovascular instability was defined either requirement of high-dose vasopressor, low cardiac output, or poor lactate clearance.

The following patient data were extracted from the electronic medical records: age, sex, preadmission diabetes medication, diabetes medication, comorbidities, age-adjusted Charlson Comorbidity Index [29], SOFA score, initial vital signs, clinical data, initial and serial laboratory results, and survival outcomes.

### Outcomes

The primary outcome was 90-day mortality. The secondary outcome was 365-day mortality.

### Statistical analysis

Continuous variables with normal distribution are presented as means and standard deviations and compared using the Student’s t-test. Continuous variables without normal distributions are presented as medians and interquartile ranges (IQRs), and compared using the Mann-Whitney U test. Categorical variables are expressed as numbers and percentages and compared using the chi-square test or Fisher’s exact test.

To evaluate the independent association between metformin administration and outcome variables, a multivariable Cox proportional hazards model was used. Variables with a p-value < 0.1 in the univariable Cox proportional hazard model (Supplementary Table 1) and well-known risk factors (based on previous studies) were entered into the multivariable Cox proportional hazard model. Kaplan-Meier analysis and log-rank tests were conducted.

Subgroup analyses were performed according to preadmission metformin administration, metformin dose, lactate level, AKI, and low-dose vasopressor at 48 h. Sensitivity analysis was performed after multiple imputations using Multivariate Imputation by Chained Equations (‘mice’ package) for cases with missing outcomes. Another sensitivity analysis was conducted after including patients with chronic kidney disease.

Statistical significance was set at *p* < 0.05. All statistical analyses were performed using R version 4.0.2 (R Foundation for Statistical Computing, Vienna, Austria).

## Results

**Fig. 1:**
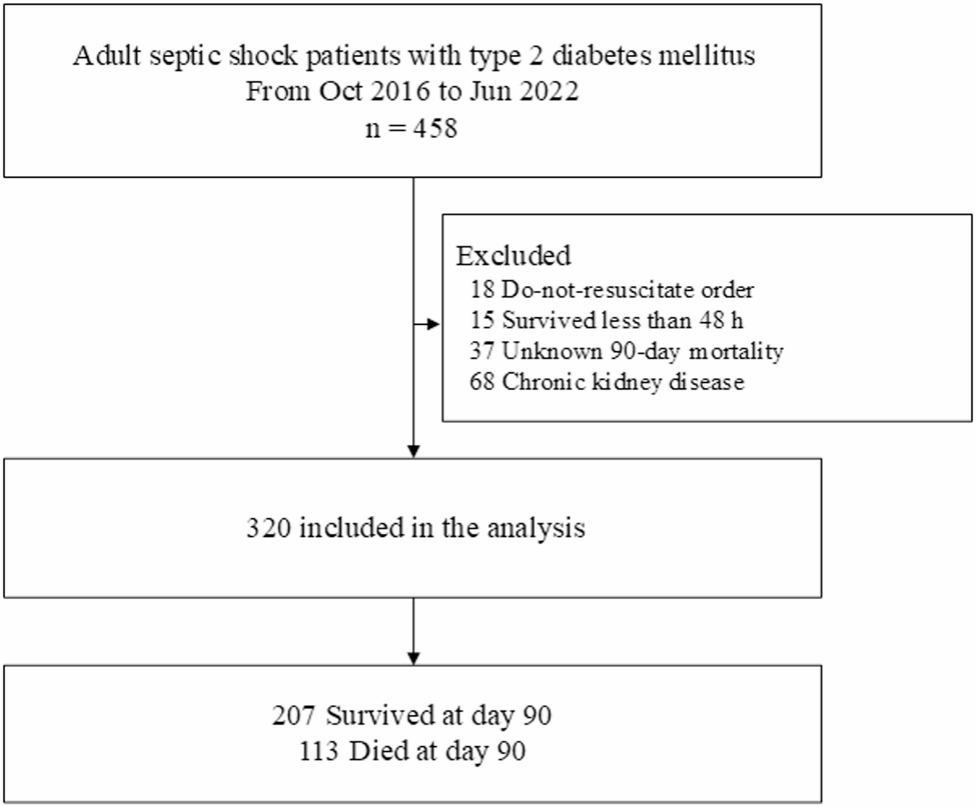
Flow chart of study population

Between October 2016 and June 2022, 458 patients with septic shock and type 2 diabetes mellitus were screened. Of the screened patients, 18 patients who had a DNR order, 15 patients who survived < 48 h, 37 patients who had unknown 90-day mortality, and 68 patients with chronic kidney disease were excluded. Finally, a total of 320 patients were included in the analysis (Fig. 1). Mean age of the study population was 72.1 ± 11.6, and mean SOFA score was 9.3 ± 2.7, and 50.9% of the cohort comprised of men and 49.1% of women. The 90-day mortality rate was 35.3% (113/320), and 365-day mortality rate was 44.5% (126/283), respectively.

Baseline characteristics according to 90-day mortality are shown in Table 1. Age, initial SOFA score, age-adjusted Charlson Comorbidity Index, respiratory rate, and lactate were significantly higher in the non-surviving group than surviving group. Infection focus as respiratory origin, malignancy, cardiovascular instability, high-dose vasopressor, low cardiac output, poor lactate clearance, and glucose intolerance requiring insulin were significantly more frequent in the non-surviving group than surviving group. Low or zero-dose vasopressor at 48 h and enteral nutrition at 48 h were significantly more frequent in the surviving group than non-surviving group. Among diabetic medications after septic shock, metformin within 48 h and metformin within 72 h were more frequently administered in the surviving group than non-surviving group (22.7% vs. 6.2%, *P* < 0.001 and 47.8% vs. 15.9%, *P* < 0.001, respectively), whereas insulin within 48 h was less frequently administered in the surviving group than non-surviving group. Baseline characteristics according to 365-day mortality (*n* = 283) are shown in Supplementary Table 2.

**Table 1:** Baseline characteristics according to 90-day mortality

Variables	Survived at day 90 (*N* = 207)	Died at day 90 (*N* = 113)	*p*-value
Sex			0.474
Men	109 (52.7%)	54 (47.8%)	
Women	98 (47.3%)	59 (52.2%)	
Age (years)	73 [62–80]	76 [67–81]	0.042
Initial SOFA score	8 [7–10]	11 [8–12]	< 0.001
**Infection focus**			< 0.001
Respiratory	63 (30.4%)	61 (54.0%)	
Gastrointestinal	27 (13.0%)	10 (8.8%)	
Biliary-pancreas	37 (17.9%)	11 (9.7%)	
Genitourinary	73 (35.3%)	23 (20.4%)	
Others	7 (3.4%)	8 (7.1%)	
**Comorbidities**			
Age-adjusted Charlson Comorbidity Index	4.6 ± 1.8	5.3 ± 1.7	0.001
Hypertension	152 (73.4%)	77 (68.1%)	0.383
Heart Disease	29 (14.0%)	17 (15.0%)	0.932
Liver Disease	14 (6.8%)	10 (8.8%)	0.649
Chronic Lung Disease	7 (3.4%)	7 (6.2%)	0.374
Stroke	43 (20.8%)	26 (23.0%)	0.747
Malignancy	32 (15.5%)	34 (30.1%)	0.003
**Preadmission Diabetic Medications**			
Metformin	86 (41.5%)	34 (30.1%)	0.057
Sulfonylurea	43 (20.8%)	20 (17.7%)	0.607
DPP4 inhibitor	63 (30.4%)	31 (27.4%)	0.664
Insulin	15 (7.2%)	5 (4.4%)	0.450
Alpha-glucosidase	4 (1.9%)	1 (0.9%)	0.802
Incretin	0 (0.0%)	0 (0.0%)	NA
Meglitinides	0 (0.0%)	2 (1.8%)	0.239
SGLT2 inhibitor	0 (0.0%)	0 (0.0%)	NA
Thiazolidinedione	6 (2.9%)	6 (5.3%)	0.437
**Initial Vital Signs**			
Systolic Blood Pressure (mmHg)	108 [89–130]	103 [83.5–123]	0.179
Diastolic Blood Pressure (mmHg)	61 [52–75]	60 [53–73]	0.778
Heart Rate (/min)	104 [88–121]	110 [98–126]	0.039
Respiratory Rate (/min)	20 [18–24]	22 [18–27]	< 0.001
Body Temperature (℃)	37.0 [36.5–38.0]	37.0 [36.0–37.0]	< 0.001
**Initial Lab**			
Lactate (mmol/L)	4.1 [3.1–6.4]	6.6 [3.9–10.3]	< 0.001
Hemoglobin (g/dL)	11.8 ± 2.5	10.6 ± 2.5	< 0.001
White blood cell (*10^3^/µL)	10.6 [6.5–16.7]	11.6 [5.4–19.5]	0.633
Platelet (*10^3^/µL)	163 [117.5–219]	157 [76–274]	0.862
Creatinine (mg/dL)	1.4 [1.0–1.9]	1.4 [0.9–2.0]	0.838
Total Bilirubin (mg/dL)	0.8 [0.5–1.4]	0.6 [0.4–1.3]	0.237
CRP (mg/dL)	10.1 [3.5–19.7]	11.7 [6.7–22.0]	0.068
Glucose (mg/dL)	183 [140.5–251.5]	189 [127–264]	0.894
HbA1c (%)*	7.2 [6.3–8.4]	6.7 [6.0–7.6]	0.079
**Initial Clinical Data**			
Acute kidney injury (any stage)	128 (61.8%)	66 (58.4%)	0.631
Cardiovascular instability (any)	92 (44.4%)	79 (69.9%)	< 0.001
High-dose vasopressor (norepinephrine-equivalent dose ≥ 0.25 µg/kg/min)	62 (30.0%)	69 (61.1%)	< 0.001
Low cardiac output	39 (18.8%)	37 (32.7%)	0.008
Poor lactate clearance	17 (8.2%)	27 (23.9%)	< 0.001
Glucose intolerance requiring insulin	43 (20.8%)	42 (37.2%)	0.001
Large aspiration	20 (9.7%)	11 (9.7%)	1.000
**Serial Clinical Data**			
Low or zero-dose vasopressor at 48 h (norepinephrine equivalent dose < 0.1 µg/kg/min)	164 (79.2%)	41 (36.3%)	< 0.001
Glucose intolerance requiring insulin at 48 h	42 (20.3%)	34 (30.1%)	0.067
Enteral nutrition at 48 h	124 (59.9%)	42 (37.2%)	< 0.001
Diabetic Medication After Septic Shock**			
Metformin within 48 h	47 (22.7%)	7 (6.2%)	< 0.001
Metformin within 72 h	99 (47.8%)	18 (15.9%)	< 0.001
Sulfonylurea within 48 h	10 (4.8%)	4 (3.5%)	0.800
DPP4 inhibitor within 48 h	28 (13.5%)	8 (7.1%)	0.119
Insulin within 48 h	94 (45.4%)	68 (60.2%)	0.016
Alpha-glucosidase within 48 h	1 (0.5%)	0 (0.0%)	1.000
Incretin within 48 h	0 (0.0%)	0 (0.0%)	NA
Meglitinides within 48 h	0 (0.0%)	1 (0.9%)	0.758
SGLT2 inhibitor within 48 h	0 (0.0%)	0 (0.0%)	NA
Thiazolidinedione within 48 h	0 (0.0%)	1 (0.9%)	0.758

Data are presented as median [interquartile range], mean ± standard deviation, or number (%), as appropriate

Abbreviations: SOFA, sequential organ failure assessment; CRP, C-reactive protein; DPP4, dipeptidyl peptidase 4; SGLT2, sodium glucose cotransporter 2

*N = 176

**Diabetic medication within 48 h after septic shock, except metformin within 72 h

The baseline characteristics according to metformin administration within 48 h are shown in Table 2. Initial SOFA score was significantly lower in metformin administration within 48 h group than no metformin group. Preadmission metformin was more frequently used in metformin administration within 48 h group than no metformin group. Age, sex, age-adjusted Charlson Comorbidity Index, initial vital signs (except for body temperature), lactate, creatinine, CRP, and HbA1c were not significantly different between the groups. Lactate and creatinine on hospital day 2 and 3, cardiovascular instability, high-dose vasopressor, low cardiac output, poor lactate clearance, glucose intolerance requiring insulin, and large aspiration were not significantly different between the groups. Low or zero-dose vasopressor at 48 h and enteral nutrition at 48 h were significantly more frequent in metformin administration within 48 h group than no metformin group. Among the diabetic medications after septic shock, sulfonylureas within 48 h and DPP4 inhibitors within 48 h were more frequently administered in metformin administration within 48 h group than no metformin group. The 90-day mortality (13.0% vs. 39.8%, *P* < 0.001), 365-day mortality (23.3% vs. 48.3%, *P* = 0.001), and in-hospital mortality (9.3% vs. 28.6%, *P* = 0.002) were significantly lower in metformin administration within 48 h group than no metformin group.

**Table 2:** Baseline characteristics according to Metformin administration within 48 h

Variables	No metformin administration within 48 h (*N* = 266)	Metformin administration within 48 h (*N* = 54)	*p*-value
Sex			0.232
Men	140 (52.6%)	23 (42.6%)	
Women	126 (47.4%)	31 (57.4%)	
Age (years)	75 [63–80]	75.5 [67–82]	0.514
Initial SOFA score	9 [8–11]	8 [7–10]	0.004
**Infection focus**			0.068
Respiratory	109 (41.0%)	15 (27.8%)	
Gastrointestinal	30 (11.3%)	7 (13.0%)	
Biliary-pancreas	39 (14.7%)	9 (16.7%)	
Genitourinary	73 (27.4%)	23 (42.6%)	
Others	15 (5.6%)	0 (0.0%)	
**Comorbidities**			
Age-adjusted Charlson Comorbidity Index	4.8 ± 1.8	5.1 ± 1.9	0.254
Hypertension	191 (71.8%)	38 (70.4%)	0.962
Heart Disease	38 (14.3%)	8 (14.8%)	1.000
Liver Disease	19 (7.1%)	5 (9.3%)	0.799
Chronic Lung Disease	13 (4.9%)	1 (1.9%)	0.529
Stroke	58 (21.8%)	11 (20.4%)	0.958
Malignancy	53 (19.9%)	13 (24.1%)	0.615
**Preadmission Diabetic Medications**			
Metformin	87 (32.7%)	33 (61.1%)	< 0.001
Sulfonylurea	51 (19.2%)	12 (22.2%)	0.744
DPP4 inhibitor	72 (27.1%)	22 (40.7%)	0.065
Insulin	17 (6.4%)	3 (5.6%)	1.000
Alpha-glucosidase	5 (1.9%)	0 (0.0%)	0.679
Incretin	0 (0.0%)	0 (0.0%)	NA
Meglitinides	1 (0.4%)	1 (1.9%)	0.758
SGLT2 inhibitor	0 (0.0%)	0 (0.0%)	NA
Thiazolidinedione	10 (3.8%)	2 (3.7%)	1.000
**Initial Vital Signs**			
Systolic Blood Pressure (mmHg)	105 [84–123]	109.5 [92–135]	0.122
Diastolic Blood Pressure (mmHg)	60 [52–73]	63.5 [54–77]	0.198
Heart Rate (/min)	106 [90–123]	104 [89–121]	0.599
Respiratory Rate (/min)	20 [18–24]	20 [18–22]	0.150
Body Temperature (℃)	37.0 [36.0–38.0]	37.5 [37.0–38.0]	0.021
**Initial Lab**			
Lactate (mmol/L)	4.8 [3.2–8.0]	3.7 [3.1–5.9]	0.051
Hemoglobin (g/dL)	11.4 ± 2.6	11.0 ± 2.2	0.270
White blood cell (*10^3^/µL)	11.2 [6.2–19.0]	9.7 [5.1–13.3]	0.150
Platelet (*10^3^/µL)	159.5 [104–235]	174 [119–242]	0.442
Creatinine (mg/dL)	1.4 [1.0–2.0]	1.4 [0.9–1.6]	0.073
Total Bilirubin (mg/dL)	0.7 [0.4–1.3]	0.6 [0.4–1.5]	0.413
CRP (mg/dL)	10.9 [4.6–20.9]	10.3 [3.8–17.3]	0.534
Glucose (mg/dL)	186.5 [130–262]	182 [156–241]	0.843
HbA1c (%)*	7.0 [6.2–8.1]	7.0 [6.3–8.2]	0.879
**Serial Lab**			
Hospital day 2**			
Lactate (mmol/L)	2.6 [1.7–4.5]	2.2 [1.3–2.8]	0.059
Creatinine (mg/dL)	1.2 [0.8–2.0]	1.1 [0.8–1.4]	0.100
Hospital day 3***			
Lactate (mmol/L)	2.4 [1.5–5.2]	1.7 [1.2–2.1]	0.107
Creatinine (mg/dL)	1.1 [0.7–1.9]	0.9 [0.6–1.1]	0.079
**Initial Clinical Data**			
Acute kidney injury (any stage)	163 (61.3%)	31 (57.4%)	0.705
Cardiovascular instability (any)	139 (52.3%)	32 (59.3%)	0.429
High-dose vasopressor (norepinephrine-equivalent dose ≥ 0.25 µg/kg/min)	110 (41.4%)	21 (38.9%)	0.854
Low cardiac output	64 (24.1%)	12 (22.2%)	0.909
Poor lactate clearance	37 (13.9%)	7 (13.0%)	1.000
Glucose intolerance requiring insulin	72 (27.1%)	13 (24.1%)	0.776
Large aspiration	26 (9.8%)	5 (9.3%)	1.000
**Serial Clinical Data**			
Low or zero-dose vasopressor at 48 h (norepinephrine equivalent dose < 0.1 µg/kg/min)	162 (60.9%)	43 (79.6%)	0.014
Glucose intolerance requiring insulin at 48 h	68 (25.6%)	8 (14.8%)	0.129
Enteral nutrition at 48 h	125 (47.0%)	41 (75.9%)	< 0.001
**Other Diabetic Medication after septic shock**			
Sulfonylurea within 48 h	8 (3.0%)	6 (11.1%)	0.022
DPP4 inhibitor within 48 h	12 (4.5%)	24 (44.4%)	< 0.001
Insulin within 48 h	137 (51.5%)	25 (46.3%)	0.583
Alpha-glucosidase within 48 h	0 (0.0%)	1 (1.9%)	0.376
Incretin within 48 h	0 (0.0%)	0 (0.0%)	NA
Meglitinides within 48 h	0 (0.0%)	1 (1.9%)	0.376
SGLT2 inhibitor within 48 h	0 (0.0%)	0 (0.0%)	NA
Thiazolidinedione within 48 h	1 (0.4%)	0 (0.0%)	1.000
**Outcome**			
In-hospital mortality	76 (28.6%)	5 (9.3%)	0.005
90-day mortality	106 (39.8%)	7 (13.0%)	< 0.001
365-day mortality****	116 (48.3%)	10 (23.3%)	0.004

Data are presented as median [interquartile range], mean ± standard deviation, or number (%), as appropriate

Abbreviations: SOFA, sequential organ failure assessment; CRP, C-reactive protein; DPP4, dipeptidyl peptidase 4; SGLT2, sodium glucose cotransporter 2

*N = 176

**N = 199 for lactate and *N* = 309 for creatinine

***N = 125 for lactate and *N* = 278 for creatinine

****N = 283

### Multivariable Cox proportional hazard model

Administration of metformin within 48 h was independently associated with decreased 90-day mortality after adjustment of sex, initial SOFA score, infection focus, age-adjusted Charlson Comorbidity Index, preadmission metformin, vital signs, lactate, hemoglobin, sulfonylurea, DPP4 inhibitor, insulin, high-dose vasopressor, low cardiac output, poor lactate clearance, glucose intolerance requiring insulin, large aspiration, low or zero-dose vasopressor at 48 h, glucose intolerance requiring insulin at 48 h, and enteral nutrition at 48 h (adjusted hazard ratio [aHR]: 0.371, 95% confidence interval [CI]: 0.153–0.900, *P* = 0.028; Table 3 and Supplementary Table 3). Administration of metformin within 48 h was also independently associated with 90-day mortality in other models (Table 3). Administration of metformin within 48 h was independently associated with decreased 365-day mortality after adjustment (aHR 0.453, 95% CI 0.219–0.937, *P* = 0.033; Table 3).

**Table 3:** Multivariable Cox proportional hazard model

	aHR	95% CI	*p*-value
**90-day mortality**			
Metformin within 48 h			
Model 1	0.371	0.153–0.900	0.028
Model 2	0.361	0.148–0.878	0.025
Model 3	0.340	0.139–0.828	0.018
Metformin within 72 h			
Model 1	0.433	0.235–0.797	0.007
Model 2	0.431	0.234–0.793	0.007
Model 3	0.420	0.227–0.776	0.006
365-day mortality*			
Metformin within 48 h			
Model 1	0.453	0.219–0.937	0.033
Model 2	0.477	0.232–0.979	0.044
Model 3	0.468	0.226–0.973	0.042
Metformin within 72 h			
Model 1	0.450	0.264–0.767	0.003
Model 2	0.454	0.266–0.774	0.004
Model 3	0.453	0.266–0.771	0.004

*N = 283

Model 1: sex, initial SOFA score, infection focus, age-adjusted Charlson Comorbidity Index, preadmission metformin, vital signs, lactate, hemoglobin, sulfonylurea, DPP4 inhibitor, insulin, high-dose vasopressor, low cardiac output, poor lactate clearance, glucose intolerance requiring insulin, large aspiration, low or zero-dose vasopressor at 48 h, glucose intolerance requiring insulin at 48 h and enteral nutrition at 48 h were adjusted

Model 2: sex, initial SOFA score, infection focus, age-adjusted Charlson Comorbidity Index, preadmission metformin, vital signs, lactate, hemoglobin, sulfonylurea, DPP4 inhibitor, insulin, cardiovascular instability (any), glucose intolerance requiring insulin, large aspiration, low or zero-dose vasopressor at 48 h, glucose intolerance requiring insulin at 48 h and enteral nutrition at 48 h were adjusted

Model 3: sex, initial SOFA score, infection focus, age-adjusted Charlson Comorbidity Index, preadmission metformin, vital signs, lactate, hemoglobin, sulfonylurea, DPP4 inhibitor, insulin, high-dose vasopressor, low cardiac output, poor lactate clearance, glucose intolerance requiring insulin, large aspiration, AKI, malignancy, low or zero-dose vasopressor at 48 h, glucose intolerance requiring insulin at 48 h and enteral nutrition at 48 h were adjusted

Administration of metformin within 72 h was independently associated with decreased 90-day mortality after adjustment (aHR 0.433, 95% CI 0.235–0.797, *P* = 0.007; Table 3). Administration of metformin within 72 h was independently associated with decreased 365-day mortality after adjustment (aHR 0.450, 95% CI 0.264–0.767, *P* = 0.003; Table 3).

### Kaplan-Meier curve

**Fig. 2:**
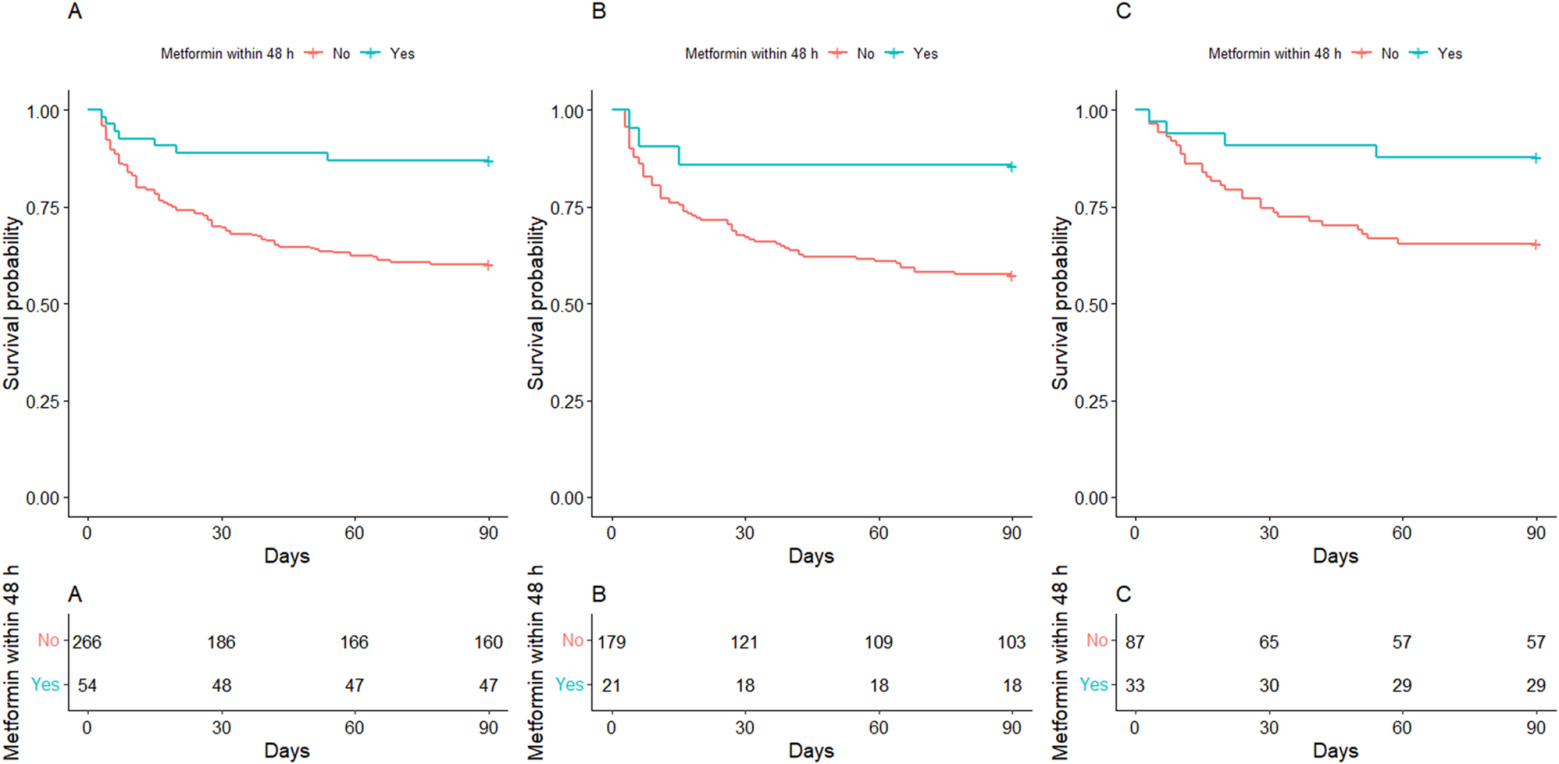
Kaplan-Meier curve. Kaplan-Meier curve according to metformin administration within 48 h (**A**). Kaplan-Meier curve in subgroup with no preadmission metformin administration (**B**). Kaplan-Meier curve in subgroup with preadmission metformin administration (**C**)

The survival rate was higher in the group with metformin administration within 48 h than no metformin group (log-rank test, *P* < 0.001; Fig. 2A).

The survival rate was higher in the group with metformin administration within 48 h than no metformin group irrespective of preadmission metformin administration (log-rank test, *P* < 0.05; Fig. 2B and C).

### Analysis according to administered metformin dose

The 90-day mortality and 365-day mortality rates were lowest in the group with metformin administration of 500–1000 mg/day (both *P* < 0.01; Supplementary Table 4).

Administration of metformin 500–1000 mg/day was independently associated with decreased 90-day and 365-day mortality after adjusting for the afore-mentioned covariables (aHR 0.311, 95% CI 0.115–0.840, *P* = 0.021 and aHR 0.384, 95% CI 0.163–0.907, *P* = 0.029, respectively; Supplementary Table 5).

In the analysis according to metformin dose, the survival rate was higher in the group with administration of metformin 500–1000 mg/day than other groups (log-rank test, *P* < 0.001; Supplementary Fig. 1).

### Subgroup analysis

The 90-day mortality was significantly lower in group with metformin administration within 48 h than no metformin group, in subgroup with AKI, in subgroup with low-dose vasopressor at 48 h, and irrespective of the lactate level (*P* < 0.05; Supplementary Table 6).

### Sensitivity analysis

In the sensitivity analysis after multiple imputations for missing outcomes, metformin administration within 48 h and 72 h were independently associated with decreased 90-day mortality after adjustment for the afore-mentioned covariables (aHR 0.396, 95% CI 0.171–0.916, *P* = 0.031 and aHR 0.461, 95% CI 0.261–0.815, *P* = 0.008, respectively).

In the sensitivity analysis after including patients with chronic kidney disease, metformin administration within 48 h and 72 h were independently associated with decreased 90-day mortality after adjustment for the afore-mentioned covariables (aHR 0.398, 95% CI 0.179–0.885, *P* = 0.023 and aHR 0.507, 95% CI 0.295–0.873, *P* = 0.014, respectively).

## Discussions

In septic shock patients with type 2 diabetes mellitus, the administration of metformin within 48 h and 72 h after septic shock was independently associated with decreased 90-day and 365-day mortality. Metformin administration was associated with decreased 90-day irrespective of preadmission metformin administration and lactate level. The results of the sensitivity analysis were similar to the main results.

Most previous studies that reported decreased mortality in sepsis patients who were administered metformin had only evaluated pre-admission or pre-morbid metformin administration and did not evaluate metformin administration after sepsis [22–25]. Only a few studies have evaluated the association between administration of metformin after sepsis or bacteremia and mortality [26, 27]. Previous studies have reported that administration of metformin after sepsis or bacteremia is significantly associated with reduced short-term mortality, which is consistent with our findings. However, those studies evaluated metformin exposure any time during hospitalization for sepsis [26, 27]. In addition, previous studies did not specifically evaluate for septic shock, did not adjust for variables that are associated with metformin administration or patients’ severity, and did not assess long-term outcomes [26, 27]. Compliance with administering metformin and the time lapse after septic shock may have a significant impact on the results. However, these factors have not yet been sufficiently investigated.

The strengths of our study are that we evaluated the administration of metformin, preadmission diabetic medications, and the time lapse after septic shock more precisely using electronic medical record data and reduced the issue of patient compliance. Furthermore, we adjusted for numerous variables related to metformin use and patient severity. We also excluded patients who survived for less than 48 h to reduce survival bias and evaluated short- and long-term survival outcomes. We conducted a sensitivity analysis and obtained similar findings, which led to robust results. The results of our study provide novel insights into diabetic medications after septic shock, such as diabetes control or potential management of sepsis, and the timing of metformin administration in patients with septic shock and type 2 diabetes mellitus.

The effect of metformin on reducing mortality in patients with sepsis may be attributed to its immunomodulatory properties, as demonstrated in both in vitro and in vivo studies [30]. Metformin has been shown to exert protective effects through both AMPK-dependent and AMPK-independent pathways, including the promotion of mitochondrial biogenesis [31] and mitophagy [32], inhibition of fatty acid synthase [33], and suppression of the NLRP3 inflammasome [34]. These effects have been reported to influence various immune cell types, including neutrophils, macrophages, and regulatory T cells [30]. In animal studies, post-insult metformin administration has been shown to mitigate sepsis-induced injury, with the interval between the insult and metformin administration ranging from 1 to 6 h [16–21].

In addition to sepsis or critically ill diseases, metformin has been associated with survival in various cohorts. In obese patients, metformin is associated with reduced mortality [8]. Metformin reduces all-cause mortality and major adverse cardiovascular events in patients with coronary artery disease and type 2 diabetes [9]. As more major cardiovascular events have been reported in patients with sepsis and diabetes [35], the beneficial effects of metformin on major cardiovascular events could have contributed to the additional beneficial effects on long-term outcomes in our study.

As preadmission metformin administration is common in patients with diabetes [7] and affects survival outcomes in sepsis [22–25], we performed a multivariable analysis adjusting for preadmission metformin administration and conducted a subgroup analysis according to preadmission metformin administration. Both analyses showed beneficial effects of metformin after septic shock, independent of preadmission metformin administration. In animal study that evaluated both prophylactic and post-insult administration of metformin [19], prophylactic metformin mitigated sepsis-induced damage, while post-insult metformin significantly improved recovery. Therefore, both preadmission metformin administration and metformin administration after septic shock might be beneficial in patients with septic shock.

Although metformin administration within 48 h and 72 h after septic shock was associated with better outcomes even after adjustment of other diabetic medications such as DPP4 inhibitors, sulfonylurea, and insulin, there might be additional benefits of other diabetic medications. DPP4 inhibitors have been reported to improve vascular dysfunction independently of their role in glucose regulation [36]. Additionally, excessive activation of K_ATP_ channels in sepsis can lead to hypotension and vascular hypo-responsiveness to catecholamines [37]; thus, sulfonylureas, which act as K_ATP_ channel blockers, may exert beneficial effects in sepsis. Moreover, given the potential benefits of maintaining well-controlled glycemia in critical illness [38], a combination of metformin with other antidiabetic agents may offer additional advantages for patients whose blood glucose levels are not adequately managed with metformin alone. The effects of DPP4 inhibitors and sulfonylurea cannot be discounted and require further studies.

The timing and dose of metformin administration after septic shock may be important. Regarding the timing of metformin in septic shock, we evaluated two timeframes, metformin administration within 48 h and 72 h after septic shock, and found that both were independently associated with reduced short- and long-term mortality. Regarding the dose of metformin in septic shock, the groups with metformin administration of 500–1000 mg/day showed lowest mortality. However, the optimal dose cannot be determined in this study, as most patients were administered with 500–1000 mg/day metformin and only a small number of patients were administered with more than 1000 mg/day metformin. In fact, all deceased patients who were administered with more than 1000 mg/day metformin developed lactic acidosis after metformin administration. Since metabolism of metformin may be altered in critically ill states, plasma metformin concentration may be an important factor. Given the insufficient evidence to determine optimal dose and timing of metformin administration in patients with septic shock, individual patient conditions should be considered when deciding on the dose and timing of metformin to maximize its beneficial effects, while closely monitoring for metformin toxicity. Further large-cohort or randomized controlled studies are warranted to establish the optimal dose and timing of metformin in patients with septic shock.

This study had several limitations. First, owing to the observational study design, there may be missed covariables. Although we conducted multivariable analysis, the missed covariables might have been unbalanced. In addition, we could only find associations, not causal relationship. Second, the number of included patients was small. The relatively small number of included patients led to a wide 95% CI. Third, the study was conducted at a single center. The results cannot be generalized to the entire population. Further multicenter studies are needed. Fourth, several patients with unknown 90-day outcomes were excluded. However, we conducted sensitivity analysis after multiple imputations and showed similar findings to the main results. Fifth, although we adjusted for other diabetic medications, there may be synergistic effects of other co-administered medications. Sixth, SGLT2 inhibitor was not evaluated because none of the patients were administered SGLT2 inhibitors within 48 h. Further studies are needed to evaluate the effects of SGLT2 inhibitors in patients with sepsis or septic shock. Seventh, metformin administration might indicate an improved clinical condition. To overcome potential selection bias, we excluded patients who survived for less than 48 h. In addition, we adjusted for variables such as cardiovascular instability, high-dose vasopressor, low cardiac output, poor lactate clearance, glucose intolerance requiring insulin, large aspiration, low or zero-dose vasopressor at 48 h, glucose intolerance requiring insulin at 48 h and enteral nutrition at 48 h, all of which are associated with patient severity and metformin use, and found benefits of metformin in patients with septic shock. Furthermore, lactate and creatinine levels on hospital day 2 and 3 were not significantly different between the groups. However, to minimize bias and ensure a balance of characteristics between groups, large-cohort studies or randomized controlled trials are warranted. Eighth, this study included only septic shock patients with type 2 diabetes mellitus. Therefore, the results cannot be generalized to septic shock patients without type 2 diabetes mellitus, warranting further study.

## Conclusion

In septic shock patients with type 2 diabetes mellitus, metformin administration within 48 h was associated with lower 90-day and 365-day mortality. While these findings suggest potential benefits of metformin administration after septic shock, further large, multicenter studies are warranted.

## Electronic supplementary material

Below is the link to the electronic supplementary material.

Supplementary Material 1

## Data Availability

The datasets used and/or analysed during the current study are available from the corresponding author on reasonable request.

## Abbreviations

DNR: Do-not-resuscitate
SOFA: Sequential organ failure assessment
SGLT2: Sodium glucose cotransporter 2
DPP4: Dipeptidyl peptidase 4
AKI: Acute kidney injury
IQR: Interquartile ranges
aHR: Adjusted hazard ratio
CI: Confidence interval
AMPK: Adenosine monophosphate-activated protein kinase
