# Supplementary material for: Association of metformin administration after septic shock with short-term and long-term survival in septic shock patients with diabetes
Source: Ann Intensive Care. 2025 May 21;15:68. doi: 10.1186/s13613-025-01490-8 (PMC12092871; doi:10.1186/s13613-025-01490-8)

**Supplementary Table 1. Univariable Cox proportional hazard model on 90-day mortality.**

| Variables | HR | 95% CI | p-value |
| --- | --- | --- | --- |
| Sex |  |  |  |
| Men | 0.853 | 0.590–1.234 | 0.398 |
| Women | Reference |  |  |
| Age (years) | 1.019 | 1.002–1.037 | 0.027 |
| Initial SOFA score | 1.190 | 1.124–1.259 | <0.001 |
| **Infection focus** |  |  |  |
| Respiratory | Reference |  |  |
| Gastrointestinal | 0.478 | 0.245–0.933 | 0.030 |
| Biliary-pancreas | 0.393 | 0.207–0.748 | 0.004 |
| Genitourinary | 0.404 | 0.250–0.653 | <0.001 |
| Others | 1.076 | 0.515–2.249 | 0.846 |
| **Comorbidities** |  |  |  |
| Age-adjusted Charlson Comorbidity Index | 1.189 | 1.083–1.305 | <0.001 |
| Hypertension | 0.848 | 0.571–1.260 | 0.415 |
| Heart Disease | 1.042 | 0.622–1.745 | 0.877 |
| Liver Disease | 1.391 | 0.727–2.662 | 0.320 |
| Chronic Lung Disease | 1.748 | 0.813–3.758 | 0.152 |
| Stroke | 1.104 | 0.712–1.711 | 0.658 |
| Malignancy | 1.870 | 1.251–2.797 | 0.002 |
| **Preadmission Diabetic Medications** |  |  |  |
| Metformin | 0.650 | 0.435–0.971 | 0.036 |
| Sulfonylurea | 0.846 | 0.522–1.372 | 0.498 |
| DPP4 inhibitor | 0.872 | 0.577––1.319 | 0.517 |
| Insulin | 0.677 | 0.276–1.659 | 0.393 |
| **Initial Vital Signs** |  |  |  |
| Systolic Blood Pressure (mmHg) | 0.996 | 0.990–1.002 | 0.199 |
| Diastolic Blood Pressure (mmHg) | 0.997 | 0.987–1.007 | 0.569 |
| Heart Rate (/min) | 1.006 | 0.999–1.012 | 0.092 |
| Respiratory Rate (/min) | 1.046 | 1.021–1.072 | <0.001 |
| Body Temperature (℃) | 0.695 | 0.605–0.798 | <0.001 |
| **Initial Lab** |  |  |  |
| Lactate (mmol/L) | 1.139 | 1.104–1.173 | <0.001 |
| Hemoglobin (g/dL) | 0.877 | 0.819–0.939 | <0.001 |
| White blood cell (*10^3^/μL) | 1.009 | 0.992–1.027 | 0.311 |
| Platelet (*10^3^/μL) | 1.001 | 0.999–1.003 | 0.175 |
| Creatinine (mg/dL) | 1.008 | 0.861–1.181 | 0.921 |
| Total Bilirubin (mg/dL) | 1.021 | 0.939–1.110 | 0.633 |
| CRP (mg/dL) | 1.012 | 0.995–1.028 | 0.178 |
| Glucose (mg/dL) | 1.001 | 0.999–1.002 | 0.266 |
| HbA1c (%)* | 0.889 | 0.760–1.041 | 0.143 |
| **Initial Clinical Data** |  |  |  |
| Acute kidney injury (any stage) | 0.910 | 0.626–1.323 | 0.623 |
| Cardiovascular instability (any) | 2.608 | 1.743–3.900 | <0.001 |
| High-dose vasopressor (norepinephrine-equivalent dose ≥0.25μg/kg/min) | 3.068 | 2.100–4.482 | <0.001 |
| Low cardiac output | 1.916 | 1.293–2.840 | 0.001 |
| Poor lactate clearance | 3.036 | 1.966–4.687 | <0.001 |
| Glucose intolerance requiring insulin | 1.886 | 1.287–2.763 | 0.001 |
| Large aspiration | 1.013 | 0.544–1.888 | 0.967 |
| **Serial Clinical Data** |  |  |  |
| Low or zero-dose vasopressor at 48 h (norepinephrine equivalent dose <0.1 μg/kg/min) | 0.211 | 0.143–0.310 | <0.001 |
| Glucose intolerance requiring insulin at 48 h | 1.474 | 0.986–2.203 | 0.059 |
| Enteral nutrition at 48 h | 0.447 | 0.305–0.655 | <0.001 |
| **Diabetic Medication After Septic Shock** |  |  |  |
| Metformin within 48 h | 0.279 | 0.130–0.599 | 0.001 |
| Metformin within 72 h | 0.259 | 0.154–0.428 | <0.001 |
| Sulfonylurea within 48 h | 0.749 | 0.276–2.031 | 0.570 |
| DPP4 inhibitor within 48 h | 0.550 | 0.267–1.128 | 0.103 |
| Insulin within 48 h | 1.567 | 1.089–2.313 | 0.016 |

Abbreviations: SOFA, sequential organ failure assessment; CRP, C-reactive protein; DPP4, dipeptidyl peptidase 4

*N=176**Supplementary Table 2. Baseline characteristics according to 365-day mortality.**

| Variables | Survived at day 365  (N=157) | Died at day 365  (N=126) | p-value |
| --- | --- | --- | --- |
| Sex |  |  | 0.760 |
| Men | 80 (51.0%) | 61 (48.4%) |  |
| Women | 77 (49.0%) | 65 (51.6%) |  |
| Age (years) | 72 [61–79.0] | 76 [67–83] | 0.007 |
| Initial SOFA score | 8 [7–10] | 10 [8–12] | <0.001 |
| **Infection focus** |  |  | <0.001 |
| Respiratory | 44 (28.0%) | 65 (51.6%) |  |
| Gastrointestinal | 21 (13.4%) | 13 (10.3%) |  |
| Biliary-pancreas | 28 (17.8%) | 12 (9.5%) |  |
| Genitourinary | 59 (37.6%) | 28 (22.2%) |  |
| Others | 5 (3.2%) | 8 (6.3%) |  |
| **Comorbidities** |  |  |  |
| Age-adjusted Charlson Comorbidity Index | 4.4 ± 1.7 | 5.3 ± 1.8 | <0.001 |
| Hypertension | 114 (72.6%) | 85 (67.5%) | 0.417 |
| Heart Disease | 20 (12.7%) | 18 (14.3%) | 0.838 |
| Liver Disease | 9 (5.7%) | 11 (8.7%) | 0.457 |
| Chronic Lung Disease | 5 (3.2%) | 7 (5.6%) | 0.492 |
| Stroke | 31 (19.7%) | 31 (24.6%) | 0.402 |
| Malignancy | 22 (14.0%) | 35 (27.8%) | 0.007 |
| **Preadmission Diabetic Medications** |  |  |  |
| Metformin | 67 (42.7%) | 38 (30.2%) | 0.041 |
| Sulfonylurea | 34 (21.7%) | 21 (16.7%) | 0.366 |
| DPP4 inhibitor | 45 (28.7%) | 34 (27.0%) | 0.858 |
| Insulin | 8 (5.1%) | 8 (6.3%) | 0.845 |
| Alpha-glucosidase | 3 (1.9%) | 1 (0.8%) | 0.776 |
| Incretin | 0 (0.0%) | 0 (0.0%) | NA |
| Meglitinides | 0 (0.0%) | 2 (1.6%) | 0.384 |
| SGLT2 inhibitor | 0 (0.0%) | 0 (0.0%) | NA |
| Thiazolidinedione | 5 (3.2%) | 6 (4.8%) | 0.709 |
| **Initial Vital Signs** |  |  |  |
| Systolic Blood Pressure (mmHg) | 109 [90–132] | 103 [83–123] | 0.108 |
| Diastolic Blood Pressure (mmHg) | 63 [52–76] | 60 [53–73] | 0.616 |
| Heart Rate (/min) | 104.5 [89–121] | 110 [94–126] | 0.209 |
| Respiratory Rate (/min) | 20 [17.5–24] | 22 [18–26] | <0.001 |
| Body Temperature (℃) | 38.0 [37.0–38.0] | 37.0 [36.0–37.4] | <0.001 |
| **Initial Lab** |  |  |  |
| Lactate (mmol/L) | 3.9 [3.0–5.8] | 6.4 [3.8–9.5] | <0.001 |
| Hemoglobin (g/dL) | 11.9 ± 2.4 | 10.5 ± 2.6 | <0.001 |
| White blood cell (*10^3^/μL) | 10.9 [6.216.4] | 11.4 [5.4–19.5] | 0.640 |
| Platelet (*10^3^/μL) | 165 [120–220] | 157 [75–268] | 0.475 |
| Creatinine (mg/dL) | 1.4 [0.9–1.9] | 1.4 [0.9–2.1] | 0.370 |
| Total Bilirubin (mg/dL) | 0.8 [0.5–1.4] | 0.6 [0.4–1.2] | 0.095 |
| CRP (mg/dL) | 10.5 [3.819.5] | 11.4 [6.0–22.0] | 0.140 |
| Glucose (mg/dL) | 187 [135–252] | 188.5 [129–273] | 0.980 |
| HbA1c (%)* | 7.3 [6.3–8.4] | 6.6 [6.0–7.7] | 0.025 |
| **Initial Clinical Data** |  |  |  |
| Acute kidney injury (any stage) | 90 (57.3%) | 75 (59.5%) | 0.801 |
| Cardiovascular instability (any) | 67 (42.7%) | 84 (66.7%) | <0.001 |
| High-dose vasopressor (norepinephrine-equivalent dose ≥0.25μg/kg/min) | 43 (27.4%) | 71 (56.3%) | <0.001 |
| Low cardiac output | 27 (17.2%) | 42 (33.3%) | 0.003 |
| Poor lactate clearance | 10 (6.4%) | 28 (22.2%) | <0.001 |
| Glucose intolerance requiring insulin | 34 (21.7%) | 45 (35.7%) | 0.013 |
| Large aspiration | 12 (7.6%) | 14 (11.1%) | 0.426 |
| **Serial Clinical Data** |  |  |  |
| Low or zero-dose vasopressor at 48 h (norepinephrine equivalent dose <0.1 μg/kg/min) | 125 (79.6%) | 50 (39.7%) | <0.001 |
| Glucose intolerance requiring insulin at 48 h | 32 (20.4%) | 36 (28.6%) | 0.144 |
| Enteral nutrition at 48 h | 99 (63.1%) | 47 (37.3%) | <0.001 |
| **Diabetic Medication After Septic Shock**** |  |  |  |
| Metformin within 48 h | 33 (21.0%) | 10 (7.9%) | 0.004 |
| Metformin within 72 h | 75 (47.8%) | 23 (18.3%) | <0.001 |
| Sulfonylurea within 48 h | 8 (5.1%) | 5 (4.0%) | 0.869 |
| DPP4 inhibitor within 48 h | 20 (12.7%) | 10 (7.9%) | 0.267 |
| Insulin within 48 h | 71 (45.2%) | 77 (61.1%) | 0.011 |
| Alpha-glucosidase within 48 h | 0 (0.0%) | 0 (0.0%) | NA |
| Incretin within 48 h | 0 (0.0%) | 0 (0.0%) | NA |
| Meglitinides within 48 h | 0 (0.0%) | 1 (0.8%) | 0.912 |
| SGLT2 inhibitor within 48 h | 0 (0.0%) | 0 (0.0%) | NA |
| Thiazolidinedione within 48 h | 0 (0.0%) | 1 (0.8%) | 0.912 |

Data are presented as median [interquartile range], mean ± standard deviation, or number (%), as appropriate.

Abbreviations: SOFA, sequential organ failure assessment; CRP, C-reactive protein; DPP4, dipeptidyl peptidase 4; SGLT2, sodium glucose cotransporter 2.

*N=158

**Diabetic medication within 48 h after septic shock, except metformin within 72 h

**Supplementary Table 3. Multivariable Cox proportional hazard model**

|  | aHR | 95% CI | p-value |
| --- | --- | --- | --- |
| **90-day mortality** |  |  |  |
| Sex | 0.916 | 0.608–1.379 | 0.674 |
| Initial SOFA score | 1.058 | 0.979–1.144 | 0.157 |
| Infection focus |  |  |  |
| Respiratory | reference |  |  |
| Gastrointestinal | 1.025 | 0.495–2.125 | 0.947 |
| Biliary-pancreas | 0.551 | 0.263–1.155 | 0.114 |
| Genitourinary | 0.542 | 0.314–0.934 | 0.028 |
| Others | 1.022 | 0.456–2.290 | 0.958 |
| Age-adjusted Charlson Comorbidity Index | 1.187 | 1.060–1.329 | 0.003 |
| Preadmission metformin | 0.940 | 0.605–1.462 | 0.784 |
| Heart Rate (/min) | 1.007 | 0.999–1.014 | 0.076 |
| Respiratory Rate (/min) | 1.014 | 0.986–1.043 | 0.342 |
| Body Temperature (℃) | 0.805 | 0.679–0.954 | 0.012 |
| Lactate (mmol/L) | 1.078 | 1.031–1.128 | 0.001 |
| Hemoglobin (g/dL) | 0.950 | 0.878–1.029 | 0.209 |
| Metformin within 48 h | 0.371 | 0.153–0.900 | 0.028 |
| Sulfonylurea within 48 h | 1.459 | 0.457–4.655 | 0.524 |
| DPP4 inhibitor within 48 h | 1.461 | 0.574–3.721 | 0.427 |
| Insulin within 48 h | 0.876 | 0.507–1.511 | 0.633 |
| High-dose vasopressor (norepinephrine-equivalent dose ≥0.25μg/kg/min) | 1.302 | 0.785–2.159 | 0.306 |
| Low cardiac output | 0.843 | 0.513–1.385 | 0.500 |
| Poor lactate clearance | 0.962 | 0.562–1.646 | 0.887 |
| Glucose intolerance requiring insulin | 2.042 | 1.140–3.660 | 0.016 |
| Large aspiration | 0.639 | 0.322–1.268 | 0.200 |
| Low or zero-dose vasopressor at 48 h (norepinephrine equivalent dose <0.1 μg/kg/min) | 0.368 | 0.226–600 | <0.001 |
| Glucose intolerance requiring insulin at 48 h | 0.773 | 0.431–1.386 | 0.387 |
| Enteral nutrition at 48 h | 0.893 | 0.570–1.397 | 0.619 |

Abbreviations: SOFA, sequential organ failure assessment; CRP, C-reactive protein; DPP4, dipeptidyl peptidase 4

**Supplementary Table 4. Outcomes according to metformin dose**

| Outcomes | No metformin | 500–1000mg/day metformin | >1000mg/day metformin | p-value |
| --- | --- | --- | --- | --- |
| 90-day mortality | 106/266 (39.8%) | 5/48 (10.4%) | 2/6 (33.3%) | <0.001 |
| 365-day mortality* | 116/240 (48.3%) | 7/38 (18.4%) | 3/5 (60.0%) | 0.002 |

Data are presented as number/total number of subgroup (%).

*N=283

**Supplementary Table 5. Multivariable Cox proportional hazard model according to metformin dose.**

|  | Multivariable Cox proportional hazard model | | |
| --- | --- | --- | --- |
|  | aHR | 95% CI | p-value |
| **90-day mortality** |  |  |  |
| No metformin | reference |  |  |
| 500–1000 mg/day | 0.311 | 0.115–0.840 | 0.021 |
| >1000 mg/day | 0.676 | 0.134–3.409 | 0.636 |
|  |  |  |  |
| **365-day mortality*** |  |  |  |
| No metformin | reference |  |  |
| 500–1000 mg/day | 0.384 | 0.163–0.907 | 0.029 |
| >1000 mg/day | 1.206 | 0.293–4.957 | 0.795 |

*N=283

**Supplementary Table 6. Subgroup analysis**

|  | No metformin administration within 48 h | Metformin administration within 48 h | p-value |
| --- | --- | --- | --- |
| **Lactate <4 mmol/L** | N=104 | N=28 |  |
| 90-day mortality | 29 (27.9%) | 2 (7.1%) | 0.041 |
| **Lactate ≥4 mmol/L** | N=162 | N=26 |  |
| 90-day mortality | 77 (47.5%) | 5 (19.2%) | 0.013 |
| **Acute kidney injury** | N=163 | N=31 |  |
| 90-day mortality | 63 (38.7%) | 3 (9.7%) | 0.004 |
| **No acute kidney injury** | N=103 | N=23 |  |
| 90-day mortality | 43 (41.7%) | 4 (17.4%) | 0.052 |
| **Low or zero-dose vasopressor at 48 h (norepinephrine equivalent dose <0.1 μg/kg/min)** | N=162 | N=43 |  |
| 90-day mortality | 39 (24.1%) | 2 (4.7%) | 0.009 |

Data are presented as number (%).

**Supplementary Figure 1.** Kaplan-Meier curve according to metformin dose.


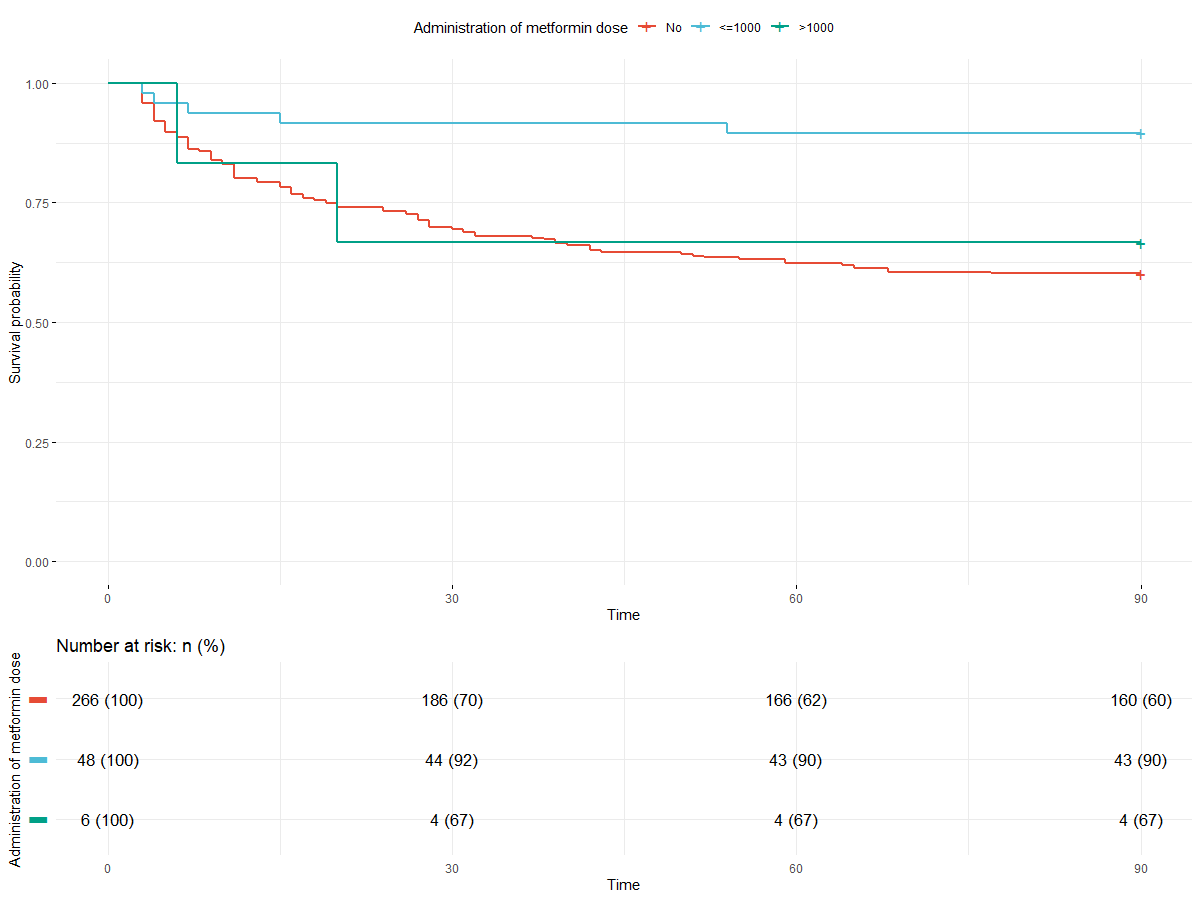

Supplement: Supplementary file 1 — Supplementary Material 1 [file 13613_2025_1490_MOESM1_ESM.docx]
